# A Dementia mortality rates dataset in Italy (2012–2019)

**DOI:** 10.1038/s41597-023-02461-z

**Published:** 2023-08-25

**Authors:** Alessandro Fania, Alfonso Monaco, Nicola Amoroso, Loredana Bellantuono, Roberto Cazzolla Gatti, Najada Firza, Antonio Lacalamita, Ester Pantaleo, Sabina Tangaro, Alena Velichevskaya, Roberto Bellotti

**Affiliations:** 1 Dipartimento Interateneo di Fisica M. Merlin, Universitá degli Studi di Bari Aldo Moro, Via G. Amendola 173, Bari, 70125 Italy; 2 Sezione di Bari, Istituto Nazionale di Fisica Nucleare (INFN), Via A. Orabona 4, Bari, 70125 Italy; 3 Dipartimento di Farmacia - Scienze del Farmaco, Universitá degli Studi di Bari Aldo Moro, Via A. Orabona 4, Bari, 70125 Italy; 4 Dipartimento di Biomedicina Traslazionale e Neuroscienze (DiBraiN), Universitá degli Studi di Bari Aldo Moro, Piazza G. Cesare 11, Bari, 70124 Italy; 5 Department of Biological Sciences, Geological and Environmental (BiGeA), Alma Mater Studiorum – University of Bologna, Piazza Porta S. Donato 1, Bologna, 40126 Italy; 6 Dipartimento di Economia e Finanza, Universitá degli Studi di Bari Aldo Moro, Largo Abbazia S. Scolastica, Bari, 70124 Italy; 7 Catholic University Our Lady of Good Counsel, Rr. Dritan Hoxha 123, Laprake, Tirana, 1031 Albania; 8 Dipartimento di Scienze del Suolo, della Pianta e degli Alimenti, Universitá degli Studi di Bari Aldo Moro, Via G. Amendola 165/a, Bari, 70126 Italy; 9 Biological Institute, Tomsk State University, Lenin Ave., 36, Tomsk, 634050 Russia

**Keywords:** Alzheimer's disease, Epidemiology

## Abstract

Dementia is on the rise in the world population and has been defined by the World Health Organization as a global public health priority. In Italy, according to demographic projections, in 2051 there will be 280 elderly people for every 100 young people, with an increase in all age-related chronic diseases, including dementia. Currently the total number of patients with dementia is estimated to be over 1 million (mainly with Alzheimer’s disease (AD) and Parkinson’s disease (PD)). In-depth studies of the etiology and physiology of dementia are complicated due to the complexity of these diseases and their long duration. In this work we present a dataset on mortality rates (in the form of Standardized Mortality Ratios, SMR) for AD e PD in Italy at provincial level over a period of 8 years (2012–2019). Access to long-term, spatially detailed and ready-to-use data could favor both health monitoring and the research of new treatments and new drugs as well as innovative methodologies for early diagnosis of dementia.

## Background & Summary

The term Dementia defines a chronic, progressive syndrome affecting cognitive and functional abilities and memory^1^. In the last years, the World Health Organization (WHO) classified dementia as a public health worldwide priority with about 55 million people affected by dementia and a cumulative incidence of about 1 new case diagnosed every 3 seconds^2,3^.

WHO estimates that by 2050 the patients will exceed 139 millions. Alzheimer disease (AD) and Parkinson disease (PD) are the most common forms of dementia. Currently, there is no cure for these diseases but treatments therapeutic to relieve symptoms. Both AD and PD are fueled by aging populations and their incidences increase with age. Despite age being the strongest risk factor, most dementias, such as AD and PD, are currently idiopathic with no cause identified with certainty. This complex and multifactorial etiology of dementia leaded to examined several factors that could influence the evolution of the disease such as genetic and environmental causes (pollution, life-style, etc...).

Italy has one of the oldest populations in the World with an estimation of more than one million dementia cases^4^. According to the WHO data report published in 2020, dementia deaths in Italy represented the fifth causes (7.64% of total deaths) with AD and PD together that appeared the first cause of death among neurodegenerative diseases^5^. In recent years, the incidences of AD and PD have shown a constantly increasing average trend in Italy which can be interpreted by aging of the population, with the consequent increase in dementia. Currently in Italy there are about 600,000 AD patients and 300,000 PD patients^6^.

Modern healthcare research on multifactorial diseases such as PD and AD and dementia in general can benefit from today’s vast availability of data. Databases that include regularly collected population-based outcomes and exposure data and clinical and biological information on individuals can aid research also through the use of modern data analysis techniques such as machine and deep learning.

**Fig. 1:**
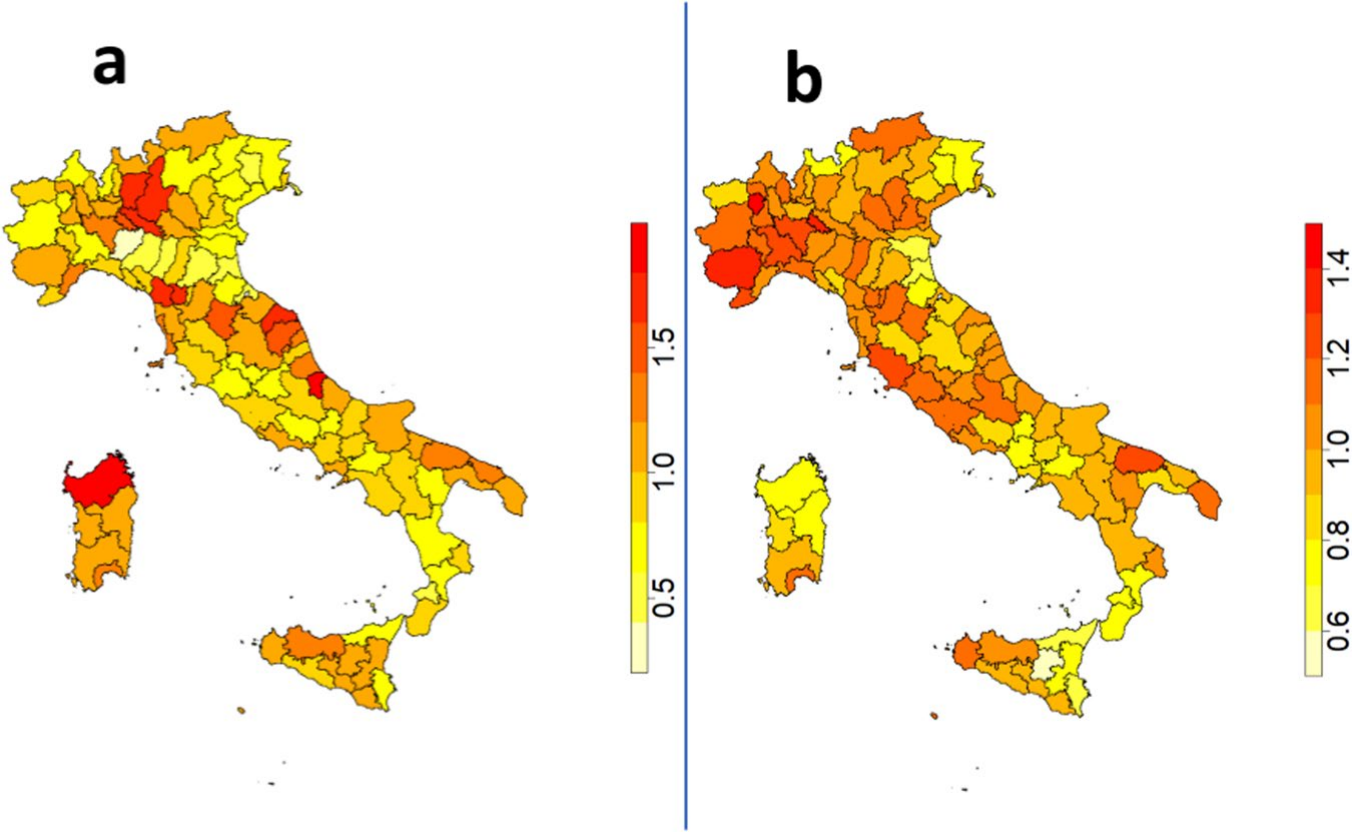
Geographical distribution of average standardized mortality rate (time windows 2012–2019) for AD and (Panel a) PD (Panel b) at provincial Italian level.

In this work we present an eight-year (2012–2019) dataset on AD and PD mortality rates in the form of Standardized Mortality Ratios (SMR)^7^ for AD and PD in Italy at provincial scale. SMR is an useful index to compare mortality rates of different populations compared to a standard. Figure 1 shows the distribution of SMR averaged over the considered time window (Panel a for AD, and panel b for PD).

The goal of this dataset is to provide an inclusive, readily accessible, and comprehensive source of information about the current situation of dementia in Italy to local and national stakeholders, policymakers, and researchers. Additionally, it aims to offer researchers with readily available data to conduct specific research. The SMR dataset can be found on the Dryad public data repository and all the source data^8^, additional information, and R codes necessary to build the dataset are available on Zenodo^9^.

## Methods

### Data source

Raw data used for the computation of SMR were collected on the Italian National Institute of Statistic website (ISTAT, http://www.istat.it/en/, last access: 26/01/2023) for a time window of 8 years (2012–2019). ISTAT is a public organization which provides statistical information about Italian territory and population. The list of variables used for computing the SMR dataset is presented in Table 1. In the following sections we detailed the single variables used for the SMR calculation.

**Table 1:** List of variables, with respective definitions and data sources, used to compute Standardized Mortality Ratio.

Symbol	Definition	Source
*M*_*i*_	Age-specific number of deaths by cause of reference population	ISTAT data warehouse^12^
*O*_*m*_	Observed number of deaths	ISTAT data warehouse^12^
*n*_*i*_	Resident population at provincial level	ISTAT data warehouse^12^
*N*_*i*_	Resident population at national level	ISTAT data warehouse^12^
${R}_{i}^{M}$	Age-specific death rates of the reference population	Calculated from Eq. (3)
*E*_*m*_	expected number of deaths by cause	Calculated from Eq. (2)
SMR	Standardized Mortality Ratio	Calculated from Eq. (1)

### Age-specific number of deaths by cause of reference population (Mi)

ISTAT provides the number of death due to a specific cause, grouped by age (*M*_*i*_). Every year, ISTAT aggregates the information coming from the registry offices, through surveys, compiled by the Civil State and physicians or necroscopes. Data are then provided on the ISTAT data warehouse, where is publicly downloadable. Specifically, on the ISTAT website, following the path Health statistics, Causes of death, Mortality by territory of residence, Cause and age, it is possible to create a data table with the required information, such as age, territory gender, causes of death, and year. In particular we considered the total number of deaths at national level by causes divided in the following 20 age groups: 0–4, 5–9, 10–14, 15–19, 20–24, 25–29, 30–34, 35–39, 40–44, 45–49, 50–54, 55–59, 60–64, 65–69, 70–74, 75–79, 80–84, 85–89, 90–94, over 95 years.

### Observed number of deaths (Om)

Mortality data are found on the ISTAT website at the path Health statistics, Causes of death, Mortality by territory of residence, Cause-prov. We selected number of deaths for Alzheimer and Parkinson diseases at provincial level (*O*_*m*_). The medical information contained in individual death certificates is encoded according to the World Health Organization’s (WHO) ICD-10 (International Statistical Classification of Diseases and Related Health Problems).

### Resident population at provincial (ni) and national levels (Ni)

The total number of resident population at provincial level (*n*_*i*_) was collected on the ISTAT data warehouse from the following path: Population and households, Intercensuary population, Reconstructed resident population -Years 2001–2019, Italy-regions-provinces. The Italian population data are estimated by ISTAT through population censuses of 2001, 2011 and 2018. The reconstruction process includes also demographic flows (births, deaths, migrations, acquisitions of citizenship). In addition to being grouped by province, the data is grouped by age, with a step of 1 year, from 0 to over 100 years. Through an aggregation process, we computed directly the national values (*N*_*i*_).

### Computation of standardized mortality ratios

In this work we computed the Standardized Mortality Ratios (SMR) for AD and PD in Italy at provincial scale. The Standardized Mortality Ratio represents the ratio between the number of deaths actually observed and the number of deaths expected, i.e. the cases that would have occurred if the study population had the same mortality as the reference population considering the different distribution by age. In other words, the SMR shows the surplus or shortage of deaths among various populations after accounting for the impacts caused by differences in age composition. The crude death rate reflects the actual situation of a total population, without considering age differences within the population. As a result, populations with a higher proportion of elderly individuals tend to exhibit higher death rates. Consequently, when analyzing trends in death rates over time, accounting for the population’s age distribution reduces the age bias. In fact, age plays a significant role in most risk factors, particularly mortality due to neurodegenerative diseases. Differences in the population structure of the geographical units of study can lead to misinterpretation of increased risk as a result of comparing unstandardized rates, especially when dealing with older populations on average. To address this, it can be used an age standardized ratio. Standardization aims to eliminate the impact of varying age distributions in populations under comparison.

The ISTAT provides the Standardized Mortality Rates only at the regional level (i.e., only for the 21 Italian regions and not for the 107 provinces). This index is quite similar to the SMR, although it differs in that it shows the standardized rate, which is the number of deaths per 10,000 inhabitants in relation to a specific reference population, rather than a ratio as in the SMR.

The expression of SMR is as follows^7^:

1 $$SMR=\frac{{O}_{m}}{{E}_{m}}$$

where *O*_*m*_ and *E*_*m*_ are the observed and the expected number of deaths by cause respectively. *E*_*m*_ has the following definition:

2 $${E}_{m}=\mathop{\sum }\limits_{i=1}^{I}{R}_{i}^{M}\ast {n}_{i}$$

in which ${R}_{i}^{M}$ is the age-specific death rates of the reference population and *n*_*i*_ is the age-specific population size for the given locality. In particular ${R}_{i}^{M}$ is defined:

3 $${R}_{i}^{M}=\frac{{M}_{i}}{{N}_{i}}$$

${R}_{i}^{M}$ is obtained dividing the number of deaths by age and cause of the reference population *M*_*i*_ with the age-specific reference population size *N*_*i*_. SMR values greater or less than 1 indicate a risk, respectively, higher or lower than that observed in the reference population. In other words, SMR values greater than 1 show a higher mortality than the Italian national one; lower values indicate a lower mortality level than the Italian national mortality. As a result SMR is an useful index to compare mortality rates of different populations compared to a standard.

## Data Record

Our dataset of Italian AD and PD mortality ratios is available for download on Dryad^8^. Specifically, the dataset includes the SMR data for AD and PD for the time window 2012–2019 at provincial level. The root folder in Dryad (“DATA”) contains 2 folders (named “AD” and “PD”) with 4 sub-folders inside each: “SMR”, “Mi”, “Ni”, “Om”. The first sub-folder includes data on SMR, while the second, the third and the fourth ones includes data on the number of death by cause, the observed number of deaths and the number of resident population at provincial level respectively. Data are provided for single province in Comma Separated Values (CSV) files. The format of CSV file for the SMR computation is the following: the first column reports the statistical code of each province; the second column contains the name of each province; the columns between the 3th and the 11th hold the value of SMR for the years 2012–2019; the columns among the 12th and 15th report four statistical calculation of SMR (mean, standard deviation, max and min values respectively). A “readme.txt” file is present in each SMR sub-folders with an explanation of the data structure.

On Zenodo^9^ is available the code in R language to reproduce the SMR computation starting from the raw data. In the main folder “Code” there are two sub-folders named “AD” and “PD” containing the scripts used for the SMR computation.

## Technical Validation

**Fig. 2:**
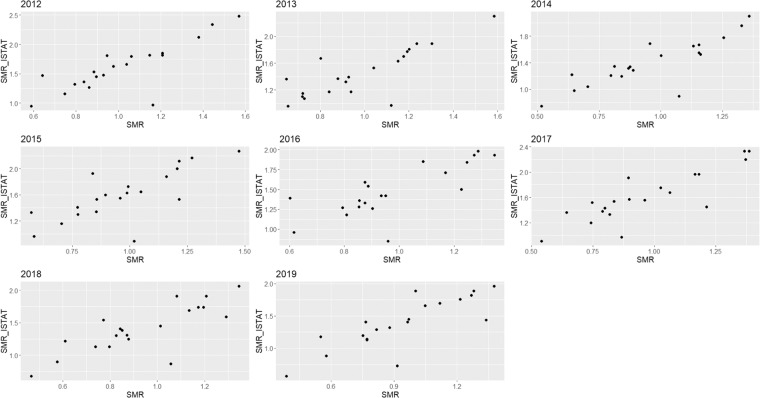
SMR_ISTAT vs. SMR scatter plot for AD and for each considered year.

**Fig. 3:**
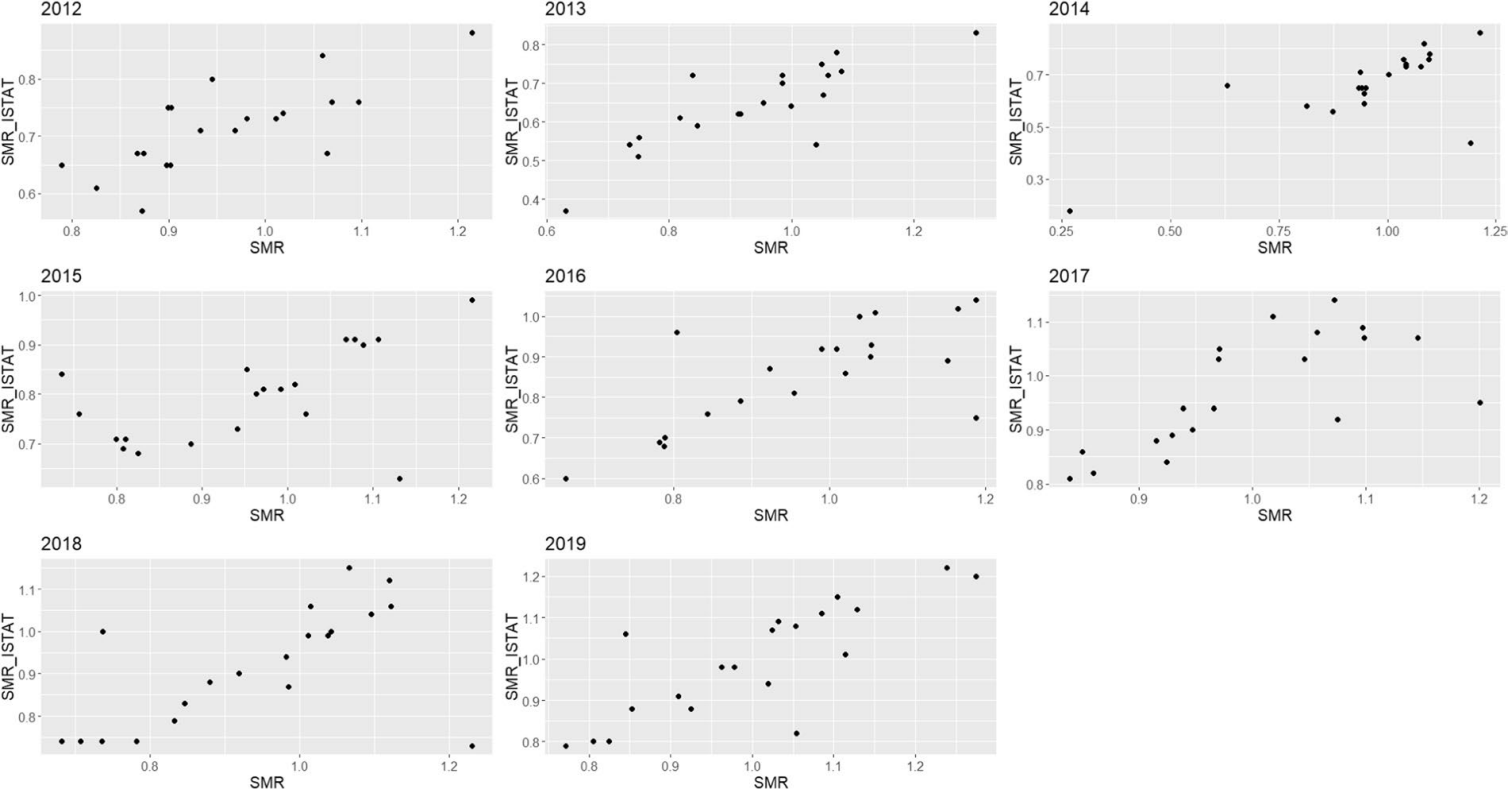
SMR_ISTAT vs. SMR scatter plot for PD and for each considered year.

In order to validate our computation of SMR, we compared it with the Standardized Mortality Rates at the regional level provided by ISTAT (SMR_ISTAT). To do this check we aggregated our provincial values of SMR to the upper level. We expected a good correlation between SMR_ISTAT and SMR values at regional level due to the common information they contain. Results of this analysis, performed each year, are showed in Figs. 2, 3 for AD and PD respectively, and reported in Table 2 in terms of Pearson correlation^10^ and the coefficient of determination (*R*^2^). The correlation between the two indices is quite strong for AD with an average value (over the considered time window) of Pearson correlation of 0.822. We found lower correlation values for PD with a mean Pearson coefficient of 0.743. In particular, the years 2015 and 2018 show the worst agreement between SMR_ISTAT and SMR.

**Table 2:** Table of performance metrics computed for each considered year.

Year	Alzheimer disease		Parkinson disease	
	Pearson Correlation	R squared	Pearson Correlation	R squared
2012	0.822	0.675	0.743	0.552
2013	0.815	0.664	0.829	0.687
2014	0.846	0.715	0.735	0.541
2015	0.752	0.566	0.563	0.317
2016	0.773	0.597	0.710	0.504
2017	0.850	0.723	0.702	0.493
2018	0.803	0.646	0.589	0.347
2019	0.819	0.670	0.813	0.661

### Data shuffling at regional level

To verify the robustness of the results reported in the previous section we applied a data shuffling procedure both on our SMR values and SMR_ISTAT at regional level. In this procedure the SMR data sets (our computation and ISTAT) for AD and PD are randomly re-sampled 100 times without replacement for each year. Then the two statistical indicators (Pearson correlation and *R*^2^) are evaluated considering these new samples. In this way we compared the agreement between SMR and SMR_ISTAT reported in the previous section and the worst case performance. In other words, we assessed how far our results are statistically from the random distribution. Our findings, reported in Table 2 present differences statistically significant with the worst case results (Table 3).

**Table 3:** Table of performance metrics after the shuffling procedure on the SMR and SMR_ISTAT values for each considered year.

Year	Alzheimer		Parkinson	
	Pearson Correlation	R squared	Pearson Correlation	R squared
2012	−0.021 ± 0.231	0.053 ± 0.06	0.001 ± 0.247	0.060 ± 0.081
2013	−0.015 ± 0.245	0.060 ± 0.071	0.022 ± 0.226	0.051 ± 0.073
2014	−0.019 ± 0.230	0.052 ± 0.070	0.008 ± 0.249	0.062 ± 0.104
2015	−0.017 ± 0.249	0.061 ± 0.078	0.018 ± 0.203	0.041 ± 0.057
2016	−0.025 ± 0.229	0.052 ± 0.073	−0.004 ± 0.256	0.065 ± 0.070
2017	−0.019 ± 0.231	0.053 ± 0.070	0.017 ± 0.229	0.052 ± 0.074
2018	−0.028 ± 0.201	0.041 ± 0.053	−0.029 ± 0.232	0.054 ± 0.064
2019	−0.015 ± 0.223	0.050 ± 0.065	0.021 ± 0.217	0.047 ± 0.068

### Slope analysis

**Fig. 4:**
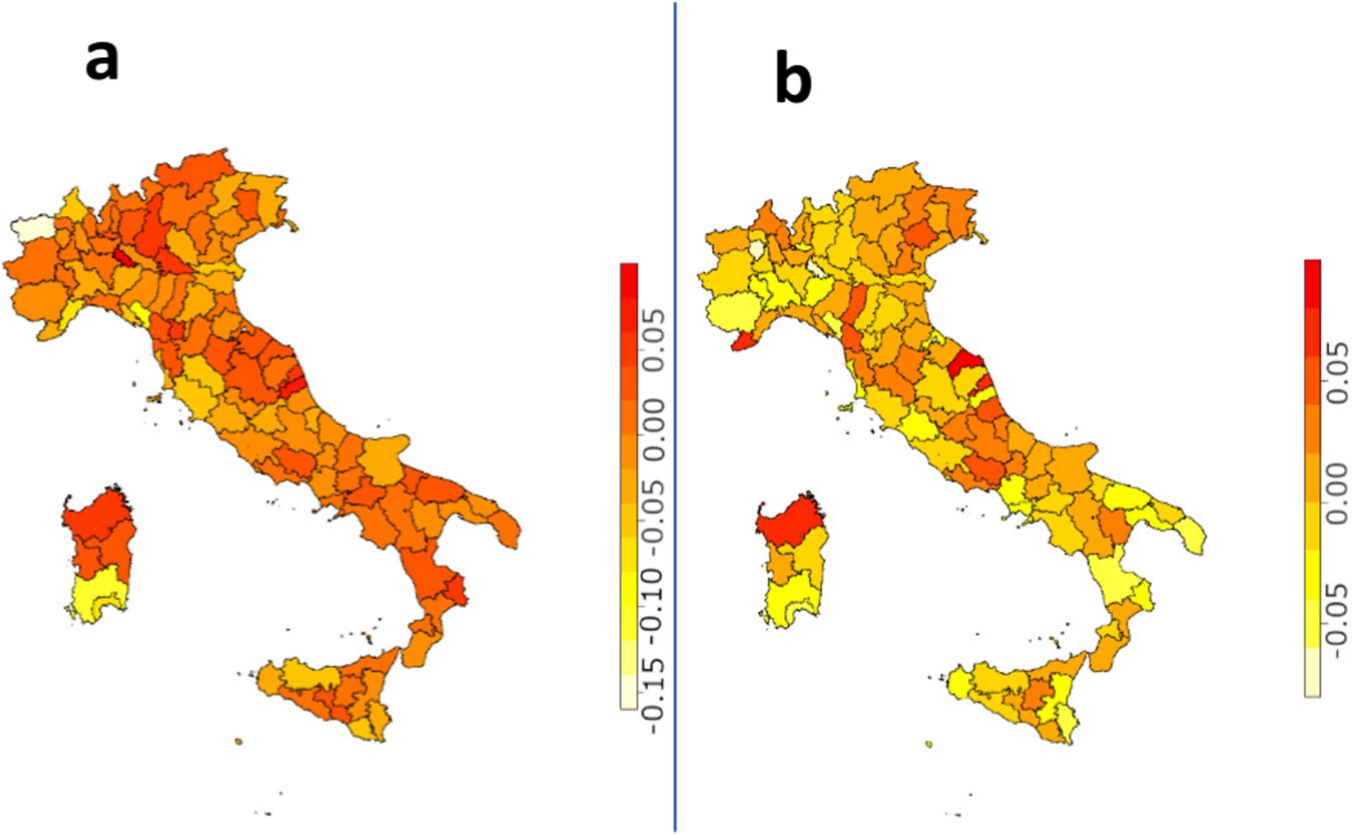
Trend of SMR values over the time period considered for each Italian province for AD and (Panel a) PD (Panel b). We considered as trend, the gradient of the line computed trough a linear regression.

After the computation of SMR, we investigated the trends of these values over the time period considered for each Italian province. In fact, using the eight years available, we carried out a linear regression from which we considered the gradient of the line. The results obtained, respectively for Alzheimer’s and Parkinson’s are shown in the panel a and b of Fig. 4. Computing the average value of the slopes over all provinces, we found slightly positve trends for AD (0.003) and PD (0.002). However, these values are very variable, with standard deviations of 0.035 and 0.030 for AD and PD respectively, highlighting a strong dependence on the SMR value of the single province. There are 56 provinces that present a positive trend for AD and 57 for PD. Eight values are too few to obtain a very robust estimation of the slope, but a future addition of data will provide a more precise assessment of the trend of AD and PD mortality over the time for the Italian territory.

## Usage Notes

This paper presents a dataset on mortality rates (in the form of Standardized Mortality Ratios, SMR) for AD e PD in Italy at provincial level between 2012 and 2019. The dataset is open to public use without limitation. The permanent storage is at^8^

## Data Availability

Data used for the computation of SMR for AD and PD at Italian provincial level are available from ISTAT (see the paragraph Data Source). We implemented the procedure described in the Methods section. Data processing was performed in R 4.2.2^11^ and the used algorithms is available on Zenodo^9^.
